# Supplementary material for: Entanglement of nanophotonic quantum memory nodes in a telecom network
Source: Nature. 2024 May 15;629(8012):573–8. doi: 10.1038/s41586-024-07252-z (PMC11096112; doi:10.1038/s41586-024-07252-z)
Supplement: Supplementary file 1 — Supplementary Information sections 1–10, Supplementary Figs. 1–7 and Supplementary Tables 1–6. [file 41586_2024_7252_MOESM1_ESM.pdf]

nature portfolio

https://doi.org/10.1038/s41586-024-07252-z

Supplementary information

Entanglement of nanophotonic quantum memory nodes in a telecom network

In the format provided by the authors and unedited

Supplementary Information for "Entanglement of Nanophotonic Quantum Memory Nodes in a Telecom Network"

I. CONTROL SEQUENCE

The control sequence for SiV control for nuclear-nuclear entanglement generation is summarized in Fig. S1. Hardware synchronization and MW- and RF-pulse generation are achieved using two Zurich Instrument HDAWG8 2.4 GSa/s AWG (HDAWG). One of the two HDAWGs acts as the controller device and is labeled control system in Fig. S1 a. It provides the central timing sequence, conditional feedback, and AOM control. The second HDAWG acts as worker device, standing by to be triggered by the controller HDAWG to play requested pulses. For more detailed sub-sequences, including electron and nuclear state measurement and initialization, see Fig. S1 b - d.

II. CAVITY-QED PARAMETERS

One of the strengths of the SiV platform is the efficient optical interface. The efficiency of the interface is due to three characteristics of the system. The first one is the strong coupling between the emitter and sub-wavelength mode of the optical cavity, which results in high cooperativity $C = \frac{4g^2}{\kappa_{\text{tot}}\gamma} > 1$. Here, g is the single-photon Rabi frequency, κ_{tot} the total cavity decay rate, and γ is the bare SiV linewidth. Decay rates and linewidths are expressed as full widths at half max (FWHM). The second one is our ability to design and fabricate overcoupled one-sided cavities. And, finally, the third one is the efficient mode matching between the cavity mode and the tapered input fiber. The reflection amplitude of light for the cavity-coupled SiV at frequency ω can be expressed as:

$$\text{Reflection}(\omega) = 1 - \frac{\kappa_{\text{in}}}{i(\omega-\omega_c) + \kappa_{\text{tot}}/2 + g^2/(i(\omega-\omega_{SiV})) + \gamma/2} \tag{S1}$$

Here, κ_{in} is the coupling rate of the cavity into the in-coupling port, and $\omega_{SiV(c)}$ is the resonance frequency of the SiV (cavity). The cavity-QED parameters and reflection data for the SiVs used in node A and node B are shown in Fig. S2. The design and fabrication of the nanophotonic cavity is described in [1, 2].

III. CONTRAST ERROR DISTILLATION

Electron-electron and nuclear-nuclear entanglement are based on state-dependent electron reflectivity. Any reflection of the nominally non-reflective state contributes to Bell state infidelities, an error source we denote contrast error. This error source contributes differently to $|\Phi_{\text{ee}}\rangle^+$ and $|\Phi_{\text{ee}}\rangle^-$, so that selecting for the heralding events for a specific Bell state allows for a higher final Bell state fidelity.

We assume that the reflective electron state has unity reflectivity, while the nominally non-reflective electron state has small non-zero reflectivity r_A (r_B) in node A (B). The photon and electrons are prepared in $|+\rangle$ and $|{\rightarrow}{\rightarrow}\rangle$, respectively. After performing the $e-\gamma$ gate at the first node, the state becomes

$$|\gamma, e_A\rangle = \frac{1}{\sqrt{2}}(|e\downarrow_{\text{e}}^A\rangle + |l\uparrow_{\text{e}}^A\rangle + r_A(|e\uparrow_{\text{e}}^A\rangle + |l\downarrow_{\text{e}}^A\rangle)). \tag{S2}$$

We use γ and $e_{A(B)}$ to denote photonic and electron states in node A (B). The photonic qubit then interacts with the second node, resulting in the state

$$\begin{aligned}|\gamma, e_A, e_B\rangle =& \frac{1}{\sqrt{2}}(|e\downarrow_{\text{e}}^A|\downarrow_{\text{e}}^B\rangle\rangle + |l\uparrow_{\text{e}}^A\uparrow_{\text{e}}^B\rangle + r_A(|e\uparrow_{\text{e}}^A\downarrow_{\text{e}}^B\rangle + |l\downarrow_{\text{e}}^A\uparrow_{\text{e}}^B\rangle) + \\ &+ r_B(|e\downarrow_{\text{e}}^A\uparrow_{\text{e}}^B\rangle + |l\uparrow_{\text{e}}^A\downarrow_{\text{e}}^B\rangle) + r_Ar_B(|e\uparrow_{\text{e}}^A\uparrow_{\text{e}}^B\rangle + |l\downarrow_{\text{e}}^A\downarrow_{\text{e}}^B\rangle)).\end{aligned} \tag{S3}$$

Then, measuring the photon in the $|\pm\rangle$ basis heralds a successful entanglement event. We define $|e_A, e_B\rangle_\pm$ as the resulting two-node state after measuring the photon in the $|\pm\rangle$ state:

$$\begin{aligned}|e_A, e_B\rangle_+ \propto& (1 + r_Ar_B)\frac{1}{\sqrt{2}}(|\uparrow_{\text{e}}^A\uparrow_{\text{e}}^B\rangle + |\downarrow_{\text{e}}^A\downarrow_{\text{e}}^B\rangle) + \\ &(r_A + r_B)\frac{1}{\sqrt{2}}(|\uparrow_{\text{e}}^A\downarrow_{\text{e}}^B\rangle + |\downarrow_{\text{e}}^A\uparrow_{\text{e}}^B\rangle),\end{aligned} \tag{S4}$$

and

$$|e_A, e_B\rangle_- \propto (1 - r_A r_B)\frac{1}{\sqrt{2}}(|{\uparrow}_e^A {\uparrow}_e^B\rangle - |{\downarrow}_e^A {\downarrow}_e^B\rangle) + \\ (r_B - r_A)\frac{1}{\sqrt{2}}(|{\uparrow}_e^A {\downarrow}_e^B\rangle + |{\downarrow}_e^A {\uparrow}_e^B\rangle). \tag{S5}$$

Here, both states are not normalized. Thus, the infidelity due to the contrast error would then be

$$\epsilon_+ = (r_A + r_B)^2/((r_A + r_B)^2 + (1 + r_A r_B)^2) \sim (r_A + r_B)^2 \\ \epsilon_- = (r_A - r_B)^2/((r_A - r_B)^2 + (1 - r_A r_B)^2) \sim (r_A - r_B)^2 \tag{S6}$$

where the approximation is done by only considering the lowest order errors in r_A, r_B. We write $r_A = |r_A|e^{i\phi_A}$ and $r_B = |r_B|e^{i\phi_B}$, and based on the cavity-QED parameters of our system get $\phi_A \approx \phi_B \approx \pi$. In this case, ϵ_- should be less than ϵ_+ and thus contrast errors in $|e_A, e_B\rangle_-$ are suppressed. For nuclear-nuclear entanglement generation, this analysis can be carried out analogously and will also result in a suppression of contrast errors for states heralded by the TDI measuring $|-\rangle$.

IV. BELL STATE FIDELITY AND ERROR CALCULATION

Both Bell state fidelity and the sample standard deviation of the Bell state fidelity can be obtained from the measured ZZ, XX, and YY correlators. The Bell state fidelity $\mathcal{F}_\rho^{|\Phi^\pm\rangle}$ of an arbitrary quantum state ρ with respect to the Bell states $|\Phi^\pm\rangle$ can be expressed as $\mathcal{F}_\rho^{|\Phi^\pm\rangle} = \frac{1}{2}P_{zz} + \frac{1}{4}P_{xx} + \frac{1}{4}P_{yy}$,[3] with

$$P_{zz} \equiv p_{zz}^{00} + p_{zz}^{11}$$
$$P_{xx} \equiv \begin{cases} p_{xx}^{01} + p_{xx}^{10} - p_{xx}^{00} - p_{xx}^{11}, & \text{for } |\Phi^-\rangle \\ p_{xx}^{00} + p_{xx}^{11} - p_{xx}^{01} - p_{xx}^{10}, & \text{for } |\Phi^+\rangle \end{cases}$$
$$P_{yy} \equiv \begin{cases} p_{yy}^{00} + p_{yy}^{11} - p_{yy}^{01} - p_{yy}^{10}, & \text{for } |\Phi^-\rangle \\ p_{yy}^{01} + p_{yy}^{10} - p_{yy}^{00} - p_{yy}^{11}, & \text{for } |\Phi^+\rangle. \end{cases}$$

Here, p_{nn}^{ij}, $nn \in \{zz, xx, yy\}$ describe the probabilities obtaining the various measurement outcomes $(i, j) \in \{0, 1\}^2$ for each measurement basis. Now, the sample variance $\sigma_\mathcal{F}^2$ of the Bell state fidelity can be expressed as $\sigma_\mathcal{F}^2 = \left(\frac{1}{2}\sigma_{zz}\right)^2 + \left(\frac{1}{4}\sigma_{xx}\right)^2 + \left(\frac{1}{4}\sigma_{yy}\right)^2$, with $\sigma^2_{nn\in\{zz,xx,yy\}}$ describing the sample variance of $P_{nn\in\{zz,xx,yy\}}$. Using the fact that $P_{nn\in\{zz,xx,yy\}}$ follows a binominal distribution, we can use the following expression of the sample variance of a binominally distributed variable with success probability p: $\sigma^2(N, p) = \frac{p(1-p)}{N}$, where N is the sample size. Noting that for P_{zz}, $p = P_{zz}$, while for P_{xx} and P_{yy}, $p = \frac{P_{nn\in\{xx,yy\}}+1}{2}$, we can finally express the sample standard deviation of the Bell state fidelity as:

$$\sigma_\mathcal{F} = \sqrt{\frac{1}{4}\frac{P_{zz}(1 - P_{zz})}{N_{zz}} + \frac{1}{16}\frac{(1 + P_{xx})(1 - P_{xx})}{N_{xx}} + \frac{1}{16}\frac{(1 + P_{yy})(1 - P_{yy})}{N_{yy}}} \tag{S7}$$

Here, $N_{nn\in\{zz,xx,yy\}}$ is the sample size for the measurements in the zz, xx, and yy basis.

V. FIDELITY BUDGETS

Table S1 and Table S2 summarize the error contributions from various sources. The individual-node errors, optical contrasts, and TDI locking performance can be directly measured, and their contributions to our target entangled states are analyzed by numerical simulation.

V.1. Electron-electron entanglement

The numerical simulation and error-budget analysis here are performed in two steps: extracting the average photon numbers per photonic qubit (μ) of experiments and running a simulation model for a given photon number. For photon number extraction, the analysis is carried out by observing the electron spin population resulting from a X basis e-γ gate: the electron spin should end up in $|\downarrow_e\rangle$ if no photon arrives, while it ends up being in an equally mixed state of $|\uparrow_e\rangle$ and $|\downarrow_e\rangle$ if ≥ 1 photon arrives. Based on the coherent state's Poisson distribution, we can then estimate μ. We subtract the infidelity resulting from imperfect MW gates, correcting for the offset observed in the μ-rate plot (Fig. S3). We then feed this photon number into a model that considers four different sources of errors: multiple photon error, imperfect optical contrast, matter-qubit operation error, and TDI locking error. The results are shown in Table S1.

V.2. Nucleus-nucleus entanglement

Here, the analysis is carried out similarly as in the above subsection, with two differences: microwave pulse errors and nuclear readout assignment errors. MW pulse errors contribute differently due to the difference between e-γ and PHONE gate. Furthermore, first-order MW errors are detected with the electron flag qubit, resulting in a lower MW error contribution. To read out nuclear states, a $\mathrm{C}_n\mathrm{NOT}_e$ gate is applied in the middle of two electron readouts, with the measured nuclear state depending on whether the electron state has flipped or not. However, using this approach, the readout fidelity is affected by $\mathrm{C}_n\mathrm{NOT}_e$ gate fidelity, which results in a nuclear readout assignment error. The results of the error analysis are shown in Table S2.

VI. EFFICIENCY BREAKDOWN

The optical losses in our system are broken down in Table S3. The losses can be seen to come from three major categories. The first is conversion loss to overcome the inhomogeneous distribution, either via visible electro-optic frequency shifting or telecom frequency conversion. The former can be improved through more complex electro-optic shifting schemes, such as serrodyning with high-bandwidth, low-voltage modulators, or shifting using coupled rings. The latter can be improved through further optimization of the conversion setup. Beyond this, the integration of direct strain control of the individual SiVs could be used to tune their transitions and through this overcome the inhomogeneous distribution without any form of photon conversion.

The second major category of losses is our spin photon gate efficiencies, which together lead to almost an order of magnitude penalty on the efficiency. The largest hit to the individual spin-photon gate efficiency is due to the intrinsic 50% penalty from carving the final spin-photon entangled state from the initial product state. This can be overcome using more complex spin-photon cavity architectures such as phase-based gates using overcoupled cavities or symmetric cavities where photons are collected both on the reflection and transmission ports.

The final category of loss is the sum of the insertion loss of the rest of the components in the system (fiber coupling, circulator, etc.). Beyond improving the insertion loss of each individual component of the system through additional optimization, the net penalty from these components can be alleviated by changing the path architecture from a serial one as used in this work, to a parallel one where a photon is split into two paths, interacts with both nodes and then is re-interfered and measured to generate entanglement, as done in previous experiments.[4–7] This parallel architecture ensures that the loss of certain components that are intrinsic to either of the two nodes (such as fiber coupling) only penalizes the overall efficiency a single time as opposed to the double penalty incurred in the serial architecture. On the other side, moving to a parallel architecture using single-photon schemes would increase the experimental complexity due to the need to stabilize two fiber paths, which is especially challenging in deployed fiber environments.

Table S3 does list the estimated photonic link efficiency, corresponding to the insertion loss of our system, and the final success probability, including the protocol-specific loss channels such as losses due to the spin-photon gates. The success probability also accounts for the efficiency hit due to the mean photon number μ of the WCS used as input in this work. To improve this aspect of the work, high-efficiency deterministic single photon sources or heralded and fed forward single photon sources made from photon-pair sources could be used.

VII. SYSTEM IMPROVEMENTS

The generated electronic Bell state error sources can be grouped into four categories: TDI locking error, optical contrast error, MW pulse error, and multi-photon error. There is no fundamental lower bound to the TDI locking error, and a straightforward improvement of the TDI design, including passive locking and environmental isolation through integration in a vacuum box, could reduce this error source to near-zero. The optical contrast error can be significantly suppressed as well. In principle, infinite contrast (and thus zero contrast error) can be achieved for any overcoupled SiV-cavity system with a cooperativity greater than one. In practice, SiV optical resonance diffusion and stray reflections can lower the contrast, though higher cooperativities mitigate these effects. With reasonable pre-selection and overcoupled SiV-cavity systems with cooperativities higher than 10, optical contrasts of 1:50 are achievable, which would reduce this error source to $\sim 4\%$ for $|\Phi^+_{\mathrm{ee}}\rangle$ and $< 1\%$ for $|\Phi^-_{\mathrm{ee}}\rangle$.

We further note that the SiV at node B was coupled to a nearby ^{13}C that caused a reduction in MW pulse fidelities. MW pulses are limited by spectral diffusion, spin-state decoherence, as well as MW driving-induced heating. Another constraint on MW pulses is the ^{29}Si state-dependent splitting of the electron MW transition.[3] This limits how short a MW pulse can be to selectively drive one transition to about 13 ns to apply $\mathrm{C_nNOT}_e$ gates, or requires very large Rabi frequencies ($\Omega \gg 33$ MHz) to effectively drive both transitions to apply an unconditional NOT_e gate. While MW gate errors of $\sim 0.1\%$ have previously been demonstrated,[3] solid state microscopic environmental noise and frequency-dependent noise differs from emitter to emitter, imposing different error limits for different emitters. However, implementing pulse optimization techniques could allow reproduceable and deterministic reduction of MW gate errors below 1%.

When using WCS as single photons, the error scales as $\sim \mu$, where μ is the average photon number of the WCS. To reach increasingly higher fidelities requires the use of increasingly lower photon numbers, which would cause a significant reduction in Bell state generation rate. If we replace the WCS with a single photon source, there would not be a need to sacrifice entanglement generation efficiency for fidelity. In this case, the multi-photon error would scale as the single photon source second order correlation at zero delay $\sim g^{(2)}(0)$. A state-of-the-art single photon source at the frequency and bandwidth of the SiV has previously shown to achieve $g^{(2)}(0) = 1.68\%$.[1] Further improving this fidelity would require improved single photon sources at the frequency and bandwidth of the SiV. Compiling all these improvements, fidelities of around 0.95 could be achievable in the near future, with even higher fidelities reachable in the longer term with higher cooperativity SiV-cavity systems and improved single photon sources.

Additionally to using a single photon source, the success rates of entanglement generation can further be improved by improving fiber coupling efficiency, with 95% coupling efficiency shown in previous work.[8] Higher cooperativity SiV-cavity systems could also enhance the cavity reflectance to 90%.[8] This could further boost the success rate by a factor of 7.7, yielding success rates of ~ 8 Hz. Access to strain tuning would remove the need for frequency shifting, further increasing the success rate by a factor of 13.5, resulting in an success rate rate of 100 Hz.

VIII. SIV PROPERTIES

The SiV properties for each node are given in Table S4. The SiVs were picked from a larger measured distribution of SiVs for a combination of desirable device cooperativity, SiV coherence time, and SiV optical stability. The T_2 coherence times of the SiV electron and nucleus can be extended with decoupling sequences of increasing length of the form XY8-N, where an XY8 sequence is repeated N times. For a given XY8 sequence, the coherence time of the nuclear spin is about 1000 times higher than for the electron spin due to the smaller nuclear magnetic dipole moment. The maximum achieved T_2 for the nuclear spin is about 2 seconds for XY8-128 (Fig. S4). More XY8 sequences do not seem to further increase the T_2, which points to a Markovian noise source limiting the coherence time to about 2 seconds. This is likely due to coupling to phonons through the electron spin.

IX. ENTANGLEMENT RESULTS ADDITIONAL DATA

Fig. S5 shows Bell state fidelities for the $|\Phi^+_{\mathrm{nn}}\rangle$ state for different nuclear decoupling times, both error-detected and raw. The success rates for this measurement are given in Table S5. Fig. S6 shows the Bell state fidelities of the raw $|\Phi^-_{\mathrm{nn}}\rangle$ state for nuclear-nuclear entanglement generation through spools of up to 40 km of low-loss telecom fiber. Fig. S7 shows the Bell state fidelities for $|\Phi^+_{\mathrm{nn}}\rangle$ for the same measurement, both error-detected and raw. The success rates for this measurement are given in Table S6. Errors due to imperfect reflectance contrast preferentially affect the $|\Phi^+_{\mathrm{nn}}\rangle$ state, see section III. The magnitude of this type of error slowly varies over time due to spectral diffusion of

the SiV, macroscopic shifts of the resonance frequency of the nanophotonic cavity, and the laser's frequency stability. These error sources contribute to the dependency of the Bell state fidelities on the fiber length shown in Fig. S7.

X. COMPATIBILITY TO TRUE SPACE-LIKE SEPARATED NODES

Our experimental configuration allows for true space-like separation of the quantum network nodes. The added complexity from the large physical separation can be divided into three categories: synchronization of quantum control hardware, frequency stabilization of lasers, and classical communication of software-timed signals. Our quantum control hardware consists of two AWGs, with one performing the role of the controller AWG, and one as the worker AWG. The worker AWG does not perform any logic operation and is activated using digital signals from the controller AWG. These digital trigger signals could be easily relayed through an additional deployed telecom fiber.[9] Physically separated nodes also result in the requirement to lock the frequency of two lasers at both nodes. This is particularly important for the laser generating the photonic time-bin qubits at node A, which should match the linewidth and frequency of the laser used to lock the TDI in node B. Such frequency stabilization could be performed by locking both lasers to stable reference cavities located in both nodes. The same strategy could be used for the 1623 nm pump laser used for QFC. Finally, software-timed communication currently is performed using our local area network (LAN) based laboratory control software.[10] Extending this LAN-based approach to use the internet or a dedicated optical-fiber network is straightforward. It is worth noting that true-space-like separation would decrease the repetition rate of the experiment, as signals indicating successful initialization of node B's nuclear and electronic spin will have to travel classically to node A before the entanglement sequence can begin (see Fig. S1), and because dynamical decoupling needs to be performed after every entanglement attempt, as opposed to only decoupling after receiving a heralding click, as is done in this work.

[1] Knall, E. *et al.* Efficient source of shaped single photons based on an integrated diamond nanophotonic system. *Physical Review Letters* **129**, 053603 (2022).

[2] Nguyen, C. T. *et al.* An integrated nanophotonic quantum register based on silicon-vacancy spins in diamond. *Physical Review B* **100**, 165428 (2019).

[3] Stas, P.-J. *et al.* Robust multi-qubit quantum network node with integrated error detection. *Science* **378**, 557–560 (2022).

[4] Humphreys, P. C. *et al.* Deterministic delivery of remote entanglement on a quantum network. *Nature* **558**, 268–273 (2018).

[5] Liu, J.-L. *et al.* A multinode quantum network over a metropolitan area. arXiv:2309.00221 (2023).

[6] Stockill, R. *et al.* Phase-tuned entangled state generation between distant spin qubits. *Physical Review Letters* **119**, 010503 (2017).

[7] van Leent, T. *et al.* Entangling single atoms over 33 km telecom fibre. *Nature* **607**, 69–73 (2022).

[8] Bhaskar, M. K. *et al.* Experimental demonstration of memory-enhanced quantum communication. *Nature* **580**, 60–64 (2020).

[9] Bersin, E. *et al.* Telecom networking with a diamond quantum memory. *PRX Quantum* **5**, 010303 (2024).

[10] Knaut, C. M. *et al.* pylabnet - Client-server, python-based laboratory software (2021).

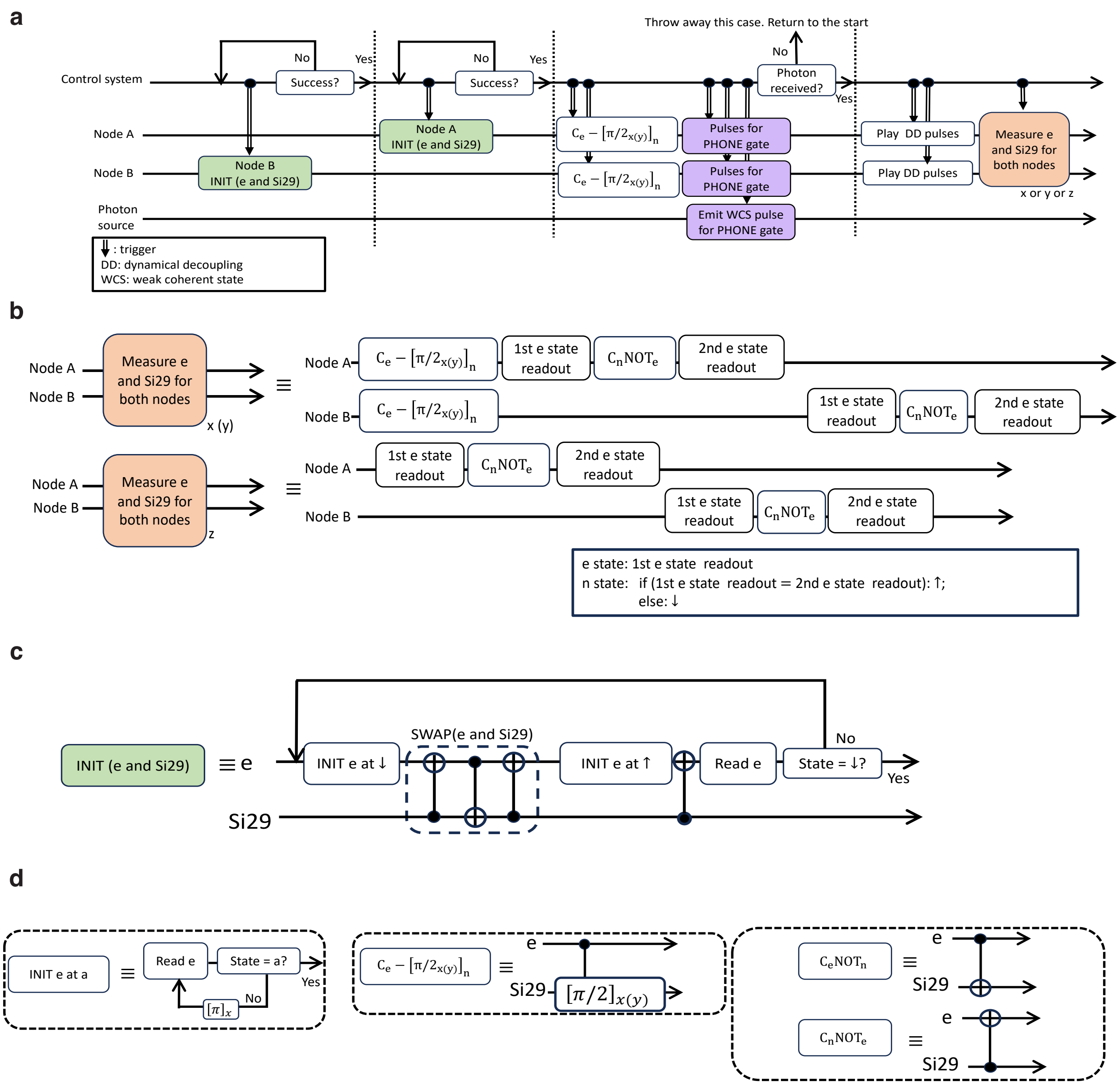

Fig. S1. **Flow chart for experimental control sequence. a.** Summarizes the overall workflow for nucleus-nucleus entanglement through PHONE gates. **b.** Detailed control sequence for the readout of the electron and the nuclear state. The electron state readout (e state readout in the figure) is performed by detecting photon numbers reflected back from nanophotonic cavities during a read laser pulse. The nuclear state is read by checking if a $C_n\mathrm{NOT}_e$-pulse flips the electron's state.[3] **c.** Detailed control sequence of initialization. The nuclear initialization is done by first initializing the electron and then swapping the electron and nuclear state. **d.** Schematic representations displayed in a.-c.

a

b

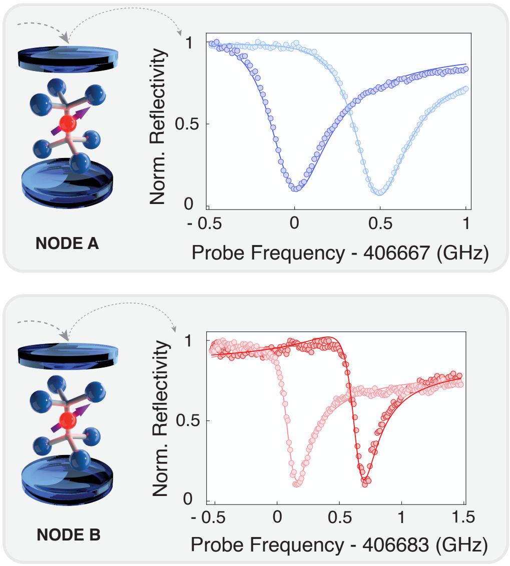

$\kappa_{tot}/2\pi$	148(1) GHz
$\kappa_{in}/2\pi$	120(4) GHz
$g/2\pi$	6.8(0) GHz
w_e	0.52 GHz
w_c	406.536(9) THz
w_{SiV}	406.667(1) THz
C	12.4(9)

$\kappa_{tot}/2\pi$	258(1) GHz
$\kappa_{in}/2\pi$	192(2) GHz
$g/2\pi$	3(1) GHz
w_e	0.53 GHz
w_c	406.641(3) THz
w_{SiV}	406.68308(7) THz
C	1.5(8)

Fig. S2. **Cavity-QED parameters a.** Node A, and **b.** Node B. Left: Reflection spectra of the cavity-QED system for two electronic spin states. Right: Values of cavity-QED parameters. ω_e is the frequency splitting between the two electronic spin-states.

Error source	Individual-node error	Error contribution $\lvert\Phi^-_{\rm ee}\rangle$	Error contribution $\lvert\Phi^+_{\rm ee}\rangle$
Microwave pulse error	2.5 ± 1.5 % (A), 3.5 ± 0.5 % (B)	12.6 ± 5.0 %	7.3 ± 2.9 %
Optical contrast error	4.3 ± 1.3 (A), 8.2 ± 1.8 % (B)	2.5 ± 0.6 %	13.0 ± 2.3 %
Multi-photon error ($\mu = 0.02$)	-	0.8 %	0.3 %
TDI locking error	-	2.0 ± 1.0 %	2.0 ± 1.0 %
Total expected error	-	16.8 ± 6.4 %	25.9 ± 5.9 %
Total observed error	-	13.8 ± 2.9 %	26.0 ± 3.5 %

Table S1. **Error budget for electron-electron entanglement.** μ: mean photon number per photonic qubit. Uncertainties for observed errors are one standard deviation. Uncertainties in the remaining rows are estimation ranges.

Error source	Individual-Node Error	Error contribution $\lvert\Phi^-_{\rm nn}\rangle^{\rm ED}$	Error contribution $\lvert\Phi^-_{\rm nn}\rangle^{\rm raw}$
Microwave pulse error	1.8 ± 0.3% (A), 7.8 ± 0.3 % (B)	≈ 0	22.1 ± 2.9 %
Optical contrast error	3.5 ± 0.5 % (A), 10.4 ± 2.1 % (B)	3.9 ± 0.9 %	3.2 ± 0.7 %
Multi-photon error ($\mu = 0.16$)	-	6.5 %	1.4 %
TDI locking error	-	2.0 ± 1.0 %	2.0 ± 1.0 %
Nuclear readout assignment error	1.5 ± 0.5 % (A), 6.0 ± 1.0 % (B)	4.3 ± 0.5 %	3.4 ± 0.4%
Total expected error	-	18.4 ± 3.1 %	37.0 ± 5.0 %
Total observed error	-	22.6 ± 5.0 %	36.7 ± 5.0 %

Table S2. **Error budget for nucleus-nucleus entanglement**. μ: mean photon number per photonic qubit. Uncertainties for observed errors are one standard deviation. Uncertainties in the remaining rows are estimation ranges.

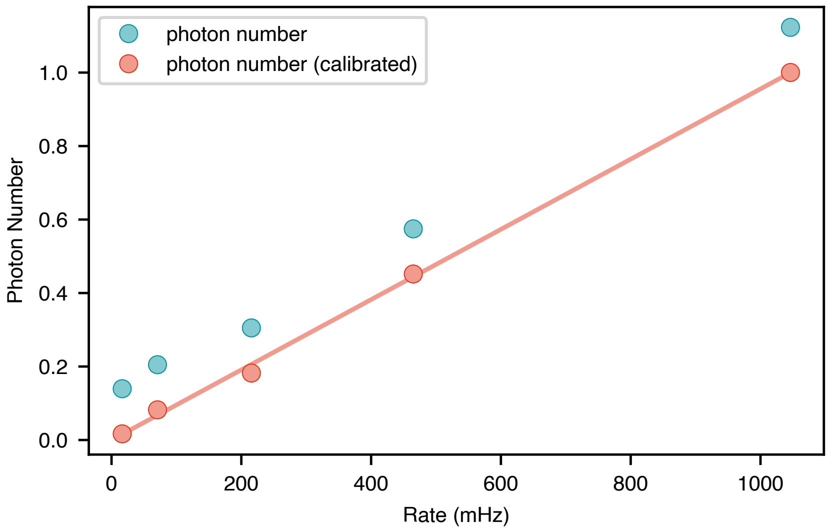

Fig. S3. **Average photon number per photonic qubit versus success rate.** The plot shows uncalibrated values (blue), and values where the MW-pulse infidelity has been compensated (red). The mean photon number is measured at node A's SiV. The solid line corresponds to the fitted linear relationship between μ and the success rate and is used for error simulations.

	Efficiency
Fiber coupling	60% (Node A) 60%^{2} (Node B)
Cavity reflectance	70% (Node A) 60% (Node B)
Node A free space setup	70 %
Visible frequency shifting	7.4 %
Telecom frequency conversion	5.4 %
Circulator	70%^{2}
SNSPD efficiency	80% and 95%
All photonic link efficiency (visible frequency shifting)	0.20 %
All photonic link efficiency (telecom frequency conversion)	0.15 %
Spin-photon gate	50%
$\|+\rangle$ / $\|-\rangle$ detection	50%
WCS mean photon number (variable)	0.1
Success probability η (visible frequency shifting)	5.0×10^{-5}
Success probability η (telecom frequency conversion)	3.7×10^{-5}

Table S3. **Estimation of photonic link efficiency and success probability in the two-node quantum network.** Efficiencies with ^{2}-superscript enter twice due to our serial network configuration. The photonic link efficiency describes the full insertion loss of our system. The success probability includes the photonic link efficiency and protocol-specific loss channels. Here, the mean photon number of the WCS has been set to a representative value of 0.1.

	Node A	Node B
τ_e	30 ns	30 ns
τ_n	∼15 µs	∼21 µs
$T_{2,e}$ XY8-1	125 µs	134 µs
$T_{2,n}$ XY8-1	339 ms	140 ms
$T_{2,n}$ XY8-128	2.11 s	2.1 s
Magnetic field	0.39 T	0.4 T

Table S4. **Node A and Node B SiV parameters.** $\tau_{e,(n)}$ is the $\mathrm{NOT}_{e,(n)}$-gate duration for the electron (nucleus).

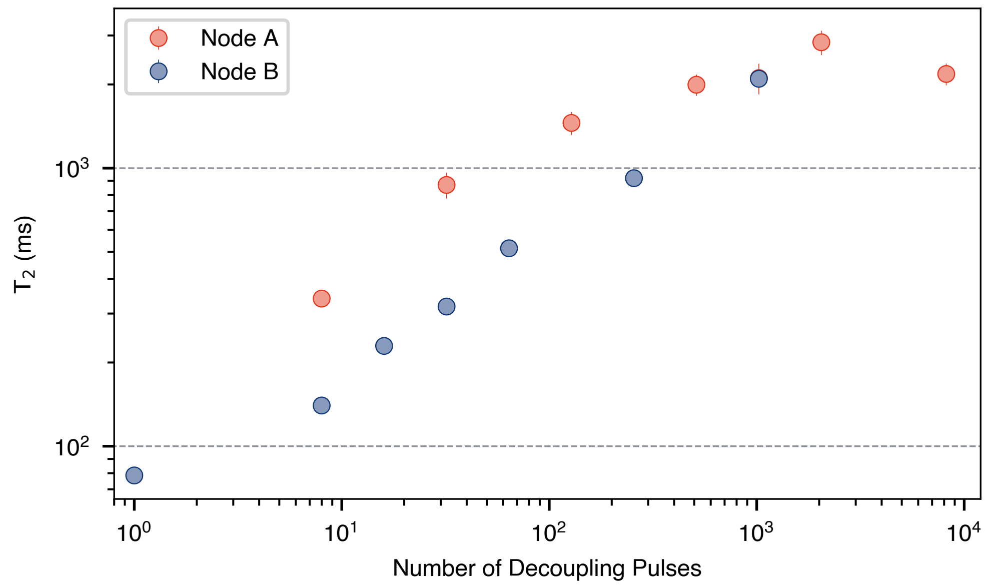

Fig. S4. **T_2 coherence times of the ^{29}Si nuclear spins.** Fitted T_2 coherence times of the ^{29}Si nuclear spin as a function of the number of decoupling pulses for node A and node B. The decoupling sequences are of the form of XY8-N, where N is the number of successive XY8 sequences. Error-bars are one s.d. Dashed lines indicate 0.1 s, and 1 s.

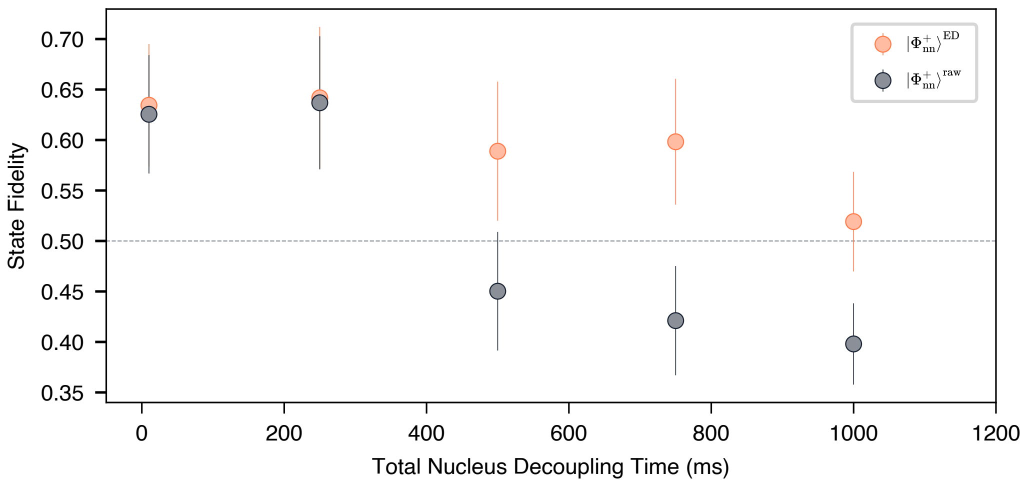

Fig. S5. $|\Phi^+_{\mathbf{nn}}\rangle$ **Bell state fidelities for decoupling experiment**. Fidelities of $|\Phi^+_{nn}\rangle$ state with (orange) and without (grey) error-detection for different total decoupling times. The dashed line indicates the classical limit. Error-bars are one s.d.

Decoupling duration (ms)	Average Rate ED	Rate $\lvert\Phi^-_{nn}\rangle^{ED}$	Rate $\lvert\Phi^+_{nn}\rangle^{ED}$
10	8.3	4.3	4.0
250	3.4	1.6	1.8
500	10.0	4.8	5.2
750	2.0	1.0	1.0
1000	6.4	3.2	3.2

Table S5. **Rates for decoupling experiment.** Summary of success rates for nuclear-nuclear entanglement generation. Rates are given in mHz.

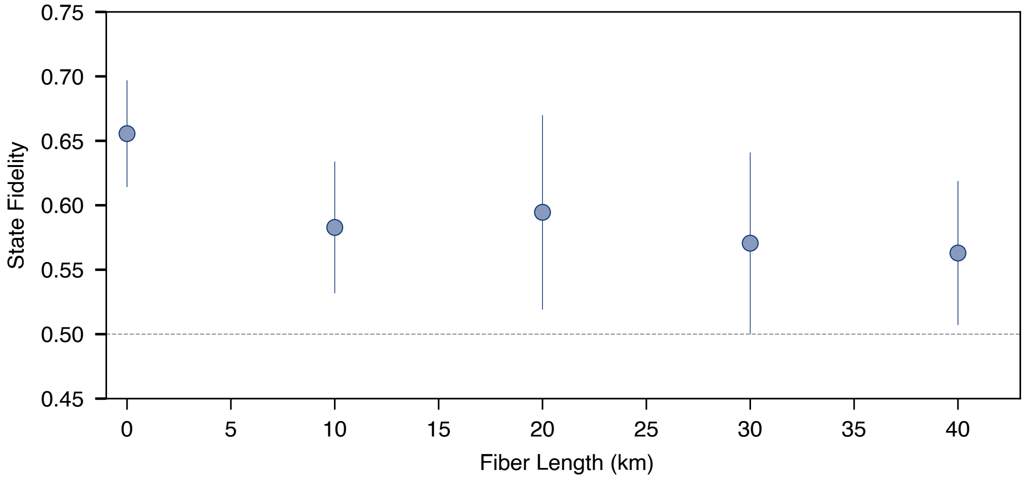

Fig. S6. $|\Phi^-_{\mathbf{nn}}\rangle$ **Bell state fidelities for telecom conversion experiment.** Bell state fidelities of $|\Phi^-_{\mathrm{nn}}\rangle^{\mathrm{raw}}$ state without error-detection for different fiber spool lengths. The dashed line indicates the classical limit. Error-bars are one s.d.

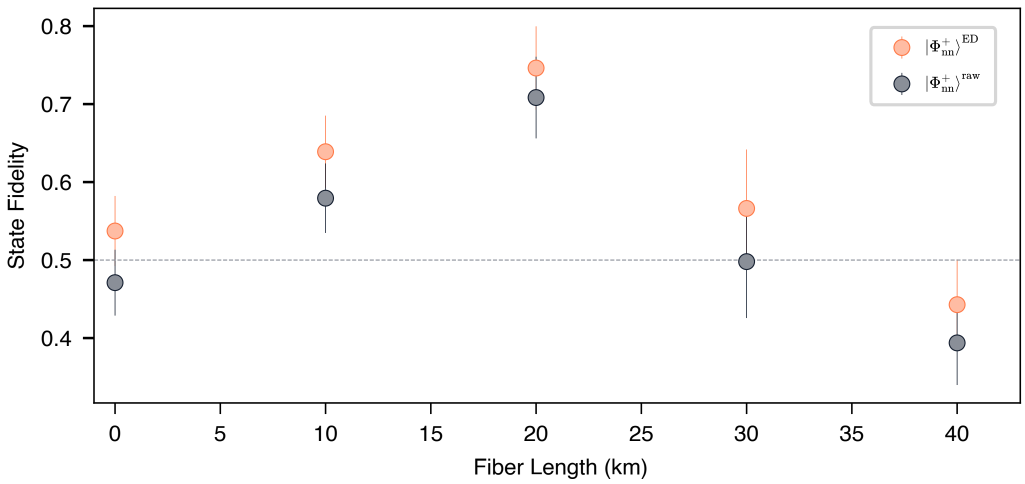

Fig. S7. $|\Phi^+_{\mathbf{nn}}\rangle$ **Bell state fidelities for telecom conversion experiment.** Bell state fidelities of $|\Phi^+_{\mathrm{nn}}\rangle$ state with (orange) and without (grey) error-detection for different fiber spool lengths. The dashed line indicates the classical limit. Error-bars are one s.d.

Fiber length (km)	Average Rate ED	Rate $\lvert\Phi^-_{\mathrm{nn}}\rangle^{\mathrm{ED}}$	Rate $\lvert\Phi^+_{\mathrm{nn}}\rangle^{\mathrm{ED}}$
0	8.2	3.7	4.5
10	5.8	2.5	3.3
20	2.9	1.1	1.8
30	5.0	2.5	2.5
40	1.5	0.7	0.8
35 (deployed)	0.23	0.10	0.14

Table S6. **Rates for telecom conversion experiment.** Summary of success rates for nuclear-nuclear entanglement generation via spools of low-loss telecom fiber and the deployed 35 km fiber loop. Rates are given in mHz.
